# Catatonia, Neuroleptic Malignant Syndrome, and Cotard Syndrome in a 22-Year-Old Woman: A Case Report

**DOI:** 10.1155/2013/452646

**Published:** 2013-09-04

**Authors:** C. Weiss, J. Santander, R. Torres

**Affiliations:** Department of Psychiatry, School of Medicine, Pontificia Universidad Católica de Chile, Apoquindo 3990, Oficina 502, Las Condes, 755-0112 Santiago, Chile

## Abstract

The following case study describes a 22-year-old woman with depression and symptoms of psychosis who developed neuroleptic malignant syndrome after using Risperidone, thus requiring life support equipment and Bromocriptine, later recovering after seven days. From a psychiatric and neurological point of view, however, the persistence of catatonic syndrome and Cotard syndrome delusions was observed, based on assertions such as “I do not have a heart,” “my heart is not beating,” “I can not breathe,” “I am breaking apart,” “I have no head” (ideas of negation) and statements about the patient being responsible for the “death of the whole world” (ideas of enormity). Brain NMR revealed leukoencephalopathy, interpreted as scar lesions caused by perinatal neurological damage, after discarding other pathologies. The patient responded well to electroconvulsive therapy after 11 sessions. Organic vulnerability to these syndromes, as well as their coexistence and clinical differentiation is discussed in the light of the data observed.

## 1. Introduction

Catatonia is a neuropsychiatric disorder that was initially described by Kahlbaum in 1874 and is characterized by motor disturbance in which patients are unable to move normally despite full physical capacity in the limbs and trunk, also it is associated with behavioral and affective disorders [1]. This condition is associated to both psychiatric and organic disorders and may impact approximately 15% of acute psychiatric patients [2]. Currently, people with affective disorders constitute the largest subgroup of catatonic patients, although catatonia has historically been associated more with schizophrenia [3]. Also linked to catatonia and even considered to be an intrinsic aspect of it is neuroleptic malignant syndrome (NMS), which involves rigidity, fever, dysautonomia and changes, in mental state caused by neuroleptics. It is considered to be a life-threatening condition, due to its high mortality index recorded to date [4, 5]. 

Cotard syndrome (CS), on the other hand, was first described by Jules Cotard in 1880 [6] as a negation delusion, which may range from the negation of the patient's body parts to that of their own existence or of the entire world. Patients with CS are typically in their mid-years or are elderly and present mood disorders, but CS is also observed in patients with schizophrenia and organic disorders [7, 8]. Very few cases of CS in adolescents and young adults have been described [9].

This study reports on the case of a young woman who, while struggling with psychotic depression, developed NMS and persistence of catatonic syndrome as well as delusions compatible to what has been described for CS in the same episode.

## 2. Clinical Case

FM is a 22-year-old woman with a background of depressive episodes, which had been successfully treated with 20 mg of Citalopram a day for 2 months at the age of 19. She requested a consultation because she had been feeling tired, sad, and anxious for 2 months, which was diagnosed as a depressive episode and was treated with Sertraline and Clonazepam. After 2 weeks of no improvement, the patient began to have trouble sleeping and paying attention, felt restless and started to have delusions, thus making statements such as “I killed my mother and grandmother,” “I killed the world,” and “I saw God and must now save the world.” In this condition the patient was sent to the emergency unit of a psychiatric hospital, where she was diagnosed with psychotic syndrome and received intravenous 5 mg of Haloperidol and 4 mg of Lorazepam for once and 3 mg of Risperidone, 50 mg of Chlorpromazine, and 10 mg of Zolpidem a day, orally. She evolved with dullness, slowing down, muteness, rigidity, and progressive psychomotor inhibition, associated with having difficulty to swallow, being less aware, and disconnected from her surroundings, until she reached a state of stupor after 4 days.

Considering such condition, she was admitted to the emergency unit of a general hospital. Her exams confirmed universal rigidity, fever, autonomic instability, leukocytosis (17,800 mm^3^), and an increase in total CK that was up to 4,839 U/L. She was diagnosed with neuroleptic malignant syndrome, thus requiring life support in the ICU as well as Bromocriptine. She stabilized after 9 days, based on normal laboratory parameters and the absence of fever and autonomic instability. Nonetheless, from a psychiatric and neurological perspective, the catatonic symptoms (stupor, muteness, and negativity) persisted and did not cease when receiving Lorazepam. Consequently, she was transferred to the psychiatric unit, where electroconvulsive therapy was initiated before 48 hours of being admitted. Due to her catatonic state, she required special medical and nurse care, and, because of her immovability and difficulty to swallow, she needed nasogastric intubation and assisted mobilization to avoid thromboembolisms. Delusional ideas compatible with CS were detected during subsequent mental examinations, relating to ideas of negation and hypochondriasis, such as “I do not have a heart,” “my heart does not beat,” “I cannot breathe,” “I'm breaking apart,” “I have no head.” Ideas of enormity were also observed through statements such as “I am responsible for the death of the whole world.” Brain NMR revealed leukoencephalopathy, interpreted as scar lesions caused by perinatal neurological damage, after discarding other pathologies. The patient responded well to electroconvulsive therapy (3 times a week), with partial response observed at the 8th session and then at the 11th session, where she showed hypomanic symptoms. The patient received maintenance ECT for 2 months, associated with the use of 20 mg of Olanzapine and up to 200 mg of Lamotrigine. Olanzapine was suspended after 4 months, and she has been euthymic to date, with important improvements in autonomy and functionality, thus having been able to maintain a stable job, a relationship, and constant psychiatric followups and partial psychotherapy sessions.

## 3. Discussion

To our understanding, the patient has an affective disorder with psychotic symptoms that, associated to the use of neuroleptics, developed into NMS, but she responded well to the medical treatment described above. Subsequently, she fell into a state of stupor, compatible with catatonic syndrome and associated with Cotard syndrome or negation delusion, from a psychopathological viewpoint, and responded well to electroconvulsive therapy. To our knowledge, this is the first report on a case with such clinical characteristics.

Catatonic syndrome may emerge from affective psychoses or psychiatric disorders, conditions within the autism spectrum, neurological disorders, medical illnesses, and reactions to medication [10, 11]. In the case of the patient described here, organic vulnerability due to structural brain alterations seemed to be at the base of this wide and severe clinical manifestation.

To this regard, there is evidence suggesting that catatonic symptoms are associated with a greater susceptibility to NMS [12, 13]. Along this line, it is important to first consider that clinical and physiopathological aspects as well as treatment responses significantly overlap [10, 14], making it difficult to distinguish between them in a clinical setting. In fact, there is a lot of controversy about whether they are distinct entities or if they are part of a continuum, being the latter favored by evidence in the literature [15, 16]. 

From a neurobiological perspective, both clinical conditions involve the same pathways (orbitofrontal and motor), which would explain the overlapping of symptoms and treatment responses. What varies is their neuromodulation. In this sense, catatonia could be characterized as a cortical psychomotor syndrome, while NMS may be considered to be a subcortical motor syndrome [15]. Regarding the patient described in this report, an overlap between NMS and catatonia of affective origin was observed, being that there are similarities between both conditions. However, with regards to NMS, for instance, distinct alterations were detectable in the laboratory as well as fever and autonomic dysfunction, which were not present when the patient was in a state of catatonia and psychotic depression. Accordingly, the literature has reported on the development of these conditions [17, 18] and on the finding that catatonic symptoms may be more likely to manifest in the presence of preexisting cerebral alterations [19]. Thus, it would be important to highlight that the case described here is the first in the literature to report an association between these syndromes in orchestration, which apparently not only share vulnerabilities, but they also have distinct characteristics as seen in mental, physical, and laboratory exams.

Furthermore, this case study contributes to the literature that has identified a trend of CS in the adolescent and young adult population as well as its greater frequency in women with bipolar disorders. The patient's positive response to electroconvulsive therapy and her mood change toward a hypomanic or manic state during treatment are also in line with what has been previously described.

The search for organic vulnerabilities is important in patients who present catatonic syndromes as well as high vulnerability to collateral effects of antipsychotics [4, 20]. Although such manifestation may be deemed to be infrequent, this case tends to reinforce the importance of considering organic vulnerability, which may explain the early appearance and intensity of the symptoms described. Likewise, the influence of the underlying medical aspects involved in triggering the distinct symptoms observed in the patient of this study has also been highlighted in previous reports [8, 11, 21], which further confirms the importance of evaluating and discarding such causes when these infrequent conditions emerge.

The pattern of signs and symptoms that characterizes catatonic syndrome is not specific to its cause. In this sense, the case described here supports what Fink et al. [3] claims in relation to the generalized belief that catatonia is only a psychiatric disorder associated with schizophrenia. This belief may limit diagnosis and treatment, being that catatonia is often caused by medical/organic triggers. In fact, the majority of the patients described by Kahlbaum had underlying organic causes, such as neurosyphilis, tuberculosis, and convulsive disorders [22]. 

With regards to Cotard syndrome, most studies that have included electroconvulsive treatment or brain resonance [21] have observed anomalies in nondominant frontal, temporal, and, occasionally, parietal lobes. This also coincides with what was detected in the patient described in this study.

From a therapeutic point of view, electroconvulsive treatment led to positive results in terms of managing acute catatonia [20, 23] and CS [9, 24, 25] and was the treatment chosen for the patient of this study despite the risks of mania or hypomania, which may be treated with atypical antipsychotics or mood stabilizers [26].
